# Detection of Astrovirus, Rotavirus C, and Hepatitis E Viral RNA in Adult and Juvenile Farmed Mink (*Neovison vison*)

**DOI:** 10.3389/fvets.2018.00132

**Published:** 2018-06-19

**Authors:** Xiao-Ting Xie, Rachel E. Macdonald, Brian Tapscott, Eva Nagy, Patricia V. Turner

**Affiliations:** ^1^Department of Pathobiology, University of Guelph Guelph, ON, Canada; ^2^Ministry of Agriculture, Food and Rural Affairs, Ontario Government Guelph, ON, Canada

**Keywords:** commercial mink, fecal virus detection, astrovirus, rotavirus, hepatitis E virus

## Abstract

Mink astrovirus (MiAstV) is known to play a major role in mink pre-weaning diarrhea, and rotavirus and hepatitis E virus (HEV) are both considered potentially zoonotic agents. These viruses are not monitored in commercial mink, and the role of these viral infections in mink health is not well understood. This study assessed the prevalence of mink astrovirus, rotavirus C, mink HEV and swine HEV in 527 pooled healthy adult female mink and mink kit fecal samples from 50 Canadian mink farms in two seasons over 4 years. Viral RNA was extracted and amplified in RT-PCR to detect mink astrovirus and HEV RdRp genes, swine HEV ORF2, and rotavirus C VP6 gene. At least 26% of all positive samples for each virus was sequenced for phylogenetic analysis. Fourteen percent of samples were astrovirus positive, while 3 and 9% of samples were rotavirus C and mink HEV positive, respectively. One adult female sample was found to be positive by PCR for swine HEV. A significantly higher number of kit samples were astrovirus- and HEV-positive compared to adult female samples (*p* = 0.01 and *p* < 0.0001, respectively). Astrovirus was detected in significantly more summer samples from adult females compared to winter samples from adult females (*p* = 0.001). The detected sequences were closely related to previously reported MiAstV, swine rotavirus C, and mink and swine HEV strains. Two astrovirus sequences were distantly related to all other detected sequences as well as previously reported MiAstVs. These results demonstrate low to moderate prevalence of the three viruses in feces from clinically healthy Canadian commercial mink, and suggest that further monitoring of these viruses may provide a better understanding of how these potentially zoonotic agents may play a role in mink enteritis and overall productivity.

## Introduction

Astrovirus, rotavirus and hepatitis E virus (HEV) have been associated with enteric and respiratory diseases in a variety of commercial animals and humans. Recent studies found that these viruses are widespread in both domestic and wild animals, and many highly divergent or novel strains have been identified to be zoonotic agents (1–7). Rotaviruses are important zoonotic pathogens that can cause significant morbidity in young livestock (2, 7, 8). Recently, human HEV infections from the consumption of contaminated, undercooked pork and rabbit products have become a significant public health concern internationally, demonstrating the need to investigate and characterize potentially zoonotic viruses in commercial animals (4, 9, 10).

In 2015, the Canadian mink industry produced over 3 million pelts, valued at $98 million (11, 12). Outbreaks of enteritis resulting in kit mortality cause significant economic loss to producers (13), and while mink enteritis is most likely the result of multiple viral and bacterial infections, the major causative agents have not been well characterized. Detection of viral RNA in healthy and diseased commercial mink may help to identify agents that may play a role in enteric disease, and also permit assessment of the risk of transmission to humans in close contact with these animals. The prevalence of astrovirus, rotavirus, and HEV on commercial Canadian mink farms has not been reported. Mink astrovirus (MiAstV) has been suggested to play a major role in mink pre-weaning diarrhea (14, 15). Both rotavirus and HEV are considered significant zoonotic agents, and are known to cause disease in humans, swine, chickens and rabbits (1, 2, 6, 7, 16). Both mink HEV and rotavirus-like viral particles have been detected in commercial mink, but neither have been previously directly associated with disease in mink (1, 7). Previously, rotavirus C (RVC) has been detected in the fecal samples of diarrheic swine, cattle, and cats (8), but has never been reported in commercial mink. Swine HEV has never been reported in commercial mink in Canada, but may be shed in the fecal matter of mink due to consumption of contaminated pork products in feed (1).

The objectives of this study were to evaluate the prevalence and persistence of mink astrovirus, mink and swine HEV, and RVC RNA on Canadian commercial mink farms. Comparisons were made between age groups, season, and year of sample collection, and the relative prevalence of the three viruses across all samples. We hypothesized that kits would have higher number of infections for all three viruses than adult female mink (15, 17). We also hypothesized that viral RNA would be detected in more samples collected in the summer, which has been shown to be true for Gram-negative bacteria in other commercial animals and humans but has not been shown to be true for astrovirus, rotavirus and HEV in mink (18, 19, 20). Additionally, based on other studies, we expected that astrovirus and mink HEV RNA would be more commonly detected in samples compared to rotavirus C RNA (1, 15, 17, 21).

## Materials and methods

### Sample collection

A total of 527 pooled fecal samples were collected from commercial mink across Ontario. A total of 50 individual farms were sampled; 43 in 2014, 45 in 2015, 43 in 2016 and 37 in 2017, representing >93% of Ontario mink farms. Over the 4-year study period, some farms pelted out, new farms were sampled, some did not participate in subsequent years or only participated in subsequent years. Fecal samples were collected in the summer season (July–October) from adult female mink (>8 months old, *n* = 183) and mink kits (<8 months old, *n* = 184) in 2014 and 2015 (*n* = 367). Only adult female samples were collected in the winter seasons (January–April) of 2016 and 2017 (*n* = 82 and *n* = 78, respectively, *n* = 160 total). The fecal samples were collected from three pens, and each sample may represent a total of three female mink or up to 15 mink kits. Pooled fecal samples were collected in sterile plastic bags, and were thoroughly mixed prior to RNA extraction. All fecal samples were cooled immediately upon collection, and were immediately transported to the laboratory and stored at −80°C until needed.

### RNA extraction and cDNA synthesis

Frozen fecal samples were thawed and a 67% fecal suspension was prepared (1 g feces in 0.5 mL PBS) and mixed using the Tissuelyser at 30 Hz for 4 min. The samples were centrifuged at 3,000 × g for 15 min, and 200 μL supernatant was removed for RNA extraction. Viral RNA extraction was performed using TRIzol reagent according to the manufacturer's instructions (ThermoFisher Scientific). Forty μL of DEPC-treated water was added to the resulting RNA pellet and incubated at 70°C for 10 min, then mixed with a pipette to resuspend the RNA. RNA concentration was measured (NanoDrop) and was held at −80°C until needed. Reverse transcription was performed using the QuantiTect Reverse-Transcriptase cDNA synthesis kit according to manufacturer's instructions (Qiagen, Mississauga, ON, Canada). RNA samples to be tested for RVC were denatured at 95°C for 5 min prior to the QuantiTect reverse transcription protocol. cDNA synthesized from viral RNA was stored at −20°C until needed.

### PCR protocols and gel imaging

PCRs were conducted using virus specific primers and annealing temperatures as described in Table 1. The mink astrovirus RdRp, mink HEV RdRp, swine HEV ORF2, and RVC VP6 genes were targeted based on previous studies on molecular detection of these viruses in mink, swine or other diarrheic animals (1, 7, 8, 17, 22). The astrovirus and HEV positive controls were synthesized based on the primer targeted regions (gBlocks Gene Fragments, IDT). A RVC positive swine was used as the positive control. PCR products were run in 2% agarose gels with 12 μL of SyberSage and a 0.1-10.0 kb DNA ladder (New England Biolabs), and visualized with ChemiDoc XRS+ (Bio-Rad). At least one positive RVC, astrovirus, mink HEV and swine HEV PCR product (if detected) from each year was sequenced to validate primer targets and to determine the variation among samples at the nucleotide level. The purified PCR product templates were sequenced using forward and reverse primers using the ABI Prism® BigDye® Terminator Cycle Sequencing Ready Reaction kit v3.1 (Applied Biosystems, Foster City, CA). GeneAmp® PCR System 9700 Thermal Cycler (Applied Biosystems) was used for cycle sequencing. The partial sequences of targeted genes detected in samples were compared to existing NCBI reference sequences. Cladograms were constructed for all sequenced samples, from which representative sequences were selected for further analysis based on clustering of the sequenced genes (23). Representative sequences from each virus detected were compared to the most closely related NCBI reference sequences (based on % identity) for all sequenced genes. All phylogenetic comparisons were conducted using Phylogeny.fr, using the MUSCLE algorithm for multiple alignment [maximum likelihood estimation (MLE)], PhyML (100 bootstrap replicates) and TreeDyn for tree building and rendering (23). Bootstrap values of all sequenced genes were compared, and one representative sequence from each cluster was used to compare to closely related viruses in a cladogram (Supplementary Table 1). Bootstrap values of 1 indicate that a node is highly supported by resampling methods.

**Table 1:** Primer conditions for the detection of astrovirus (AV), rotavirus C, and hepatitis E virus (HEV) in mink fecal samples.

**Virus**	**Primer names**	**Primer sequence**	**Product size (bp)**	**Annealing temperature (°C)**	**References**
Mink astrovirus	MA15	5′CAAATGCCTGGAAGAACAC-3′	189	52	(17)
	MA17	5′-GAGGAGTT TCAGACAGATG-3′			
Rotavirus C	RVC6-3F	5′-GTTGCATCCGTGAAGAGAATG-3′	356	59.5	(8, 22)
	C4	5′-AGCCACATAGTTCACATTTCATCC−3′			
Mink hepatitis E virus	HepE-fwd	5′-CCAGAATGGTGCTTCTATGGTGAT-3′	261	60	(1)
	HepE-rev	5′-AATTGTTCTGCGAGCTATCAAACTC-3′			
Swine hepatitis E virus	3156NF	5′-AATTATGCYCAGTAYCGRGTTG-3′	348	55	
	3157NR	5′- CCCTTRTCYTGCTGMGCATTCTC-3′			
	3158NF	5′-GTWATGCTYTGCATWCATGGCT-3′			
	3159NR	5′AGCCGACGAAATCAATTCTGTC-3′			

### Data analyses

Pearson Chi-Squared tests (JMP, SAS Institute) were used to statistically compare the prevalence of detected viral RNA between adult female mink and mink kits, between the two summer season collection periods (2014 and 2015, kits and adult female samples), as well as between the adult female samples collected in the summer and winter seasons. The False Discovery Rate (FDR) was applied for Chi-Squared tests comparing the prevalence of virus RNA in adult female mink samples across all years sampled (JMP Add-In). For all statistical tests conducted, a *p*-value ≤ 0.05 was considered significant.

## Results

Overall, of the 527 female mink and mink kit pooled fecal samples collected in 2014–2017, 14% (75/527) were positive for astrovirus RNA, 9% (48/527) were positive for HEV RNA, and 3% (14/527) were positive for RVC RNA. One adult female sample from the winter collection period of 2015 was positive for swine HEV. Of the 343 pooled female mink fecal samples collected in 2014–2017, 10% (34/343) were astrovirus RNA positive, 5% (18/343) were HEV RNA positive, and 4% (12/343) were RVC RNA positive (Table 2). Twenty-four percent (44/184) of pooled kit fecal samples collected in 2014 and 2015 were positive for astrovirus RNA, 17% (32/184) were positive for HEV RNA, and 2% (4/184) were positive for RVC RNA (Table 2). Astrovirus and HEV RNA were both detected in significantly more pooled kit samples compared to pooled female samples (*p* = 0.01 and *p* < 0.0001, respectively) in the summer samples collected in 2014 and 2015. Considering all samples collected in 2014 and 2015, astrovirus RNA was detected in a significantly higher percentage of samples in 2014 than in 2015 (*p* = 0.005). HEV and RVC RNA was detected in a higher percentage of 2015 samples than 2014 samples (*p* = 0.05 and *p* = 0.01, respectively). When comparing only the female samples from all years, astrovirus RNA was detected in a higher percentage of samples from the summer 2014 compared those from the winter 2016 (*q* = 0.02), and from winter 2017 (*q* = 0.002). When comparing only female samples, astrovirus RNA was detected in a significantly higher percentage of samples collected in the summer season compared to the winter season (*p* = 0.001). Samples from 2015 had a higher number of astrovirus RNA positive samples compared to samples from 2017 (*q* = 0.02), and samples from 2017 had a slightly higher number of RVC RNA positive samples compared to those from 2016 (*q* = 0.05).

**Table 2:** Prevalence of astrovirus (AV), rotavirus C (RVC), and hepatitis E virus (HEV) RNA in pooled female mink and kit fecal samples collected in 2014–2017.

		**Females (%)**			**Kits (%)**		
**Year**	**Season**	**AV**	**RVC**	**HEV**	**AV**	**RVC**	**HEV**
2014	Summer	18 (16/87)	0	2 (2/87)	31 (27/86)	0	13 (11/86)
2015	Summer	9 (9/96)	3 (3/96)	6 (6/96)	17 (17/98)	4 (4/98)	21 (21/98)
2016	Winter^*^	6 (5/82)	1 (1/82)	5 (4/82)	–	–	–
2017	Winter^*^	1 (1/78)	8 (6/78)	5 (4/78)	–	–	–
Total (*n* = 527):	10 (34/343)	4 (12/343)	5 (18/343)	24 (44/184)	2 (4/184)	17 (32/184)	

* *Only pooled fecal samples from adult female mink could be collected in the winter*.

In 2014, 2 adult female and 3 kit pooled fecal samples were positive for both astrovirus and HEV RNA. Two kit samples from 2015 had both astrovirus and HEV RNA, and one kit sample was positive for both RVC and HEV RNA. One adult female fecal sample in 2017 had detectable RVC and astrovirus RNA. A farm was considered positive if at least one sample was positive for any one of the screened viruses for any given year. Analysis of farm status across 50 farms showed that only one farm was RVC positive two consecutive years (2016–2017), and three farms were HEV positive for two consecutive years (2014–2015, 2015–2016, and 2016–2017). No farms were positive for RVC or HEV for more than two consecutive years. In the case of astrovirus, only one farm was positive for three consecutive years (2014–2016), while 11 farms were positive for two consecutive years (2014–2015 only).

**Figure 1:**
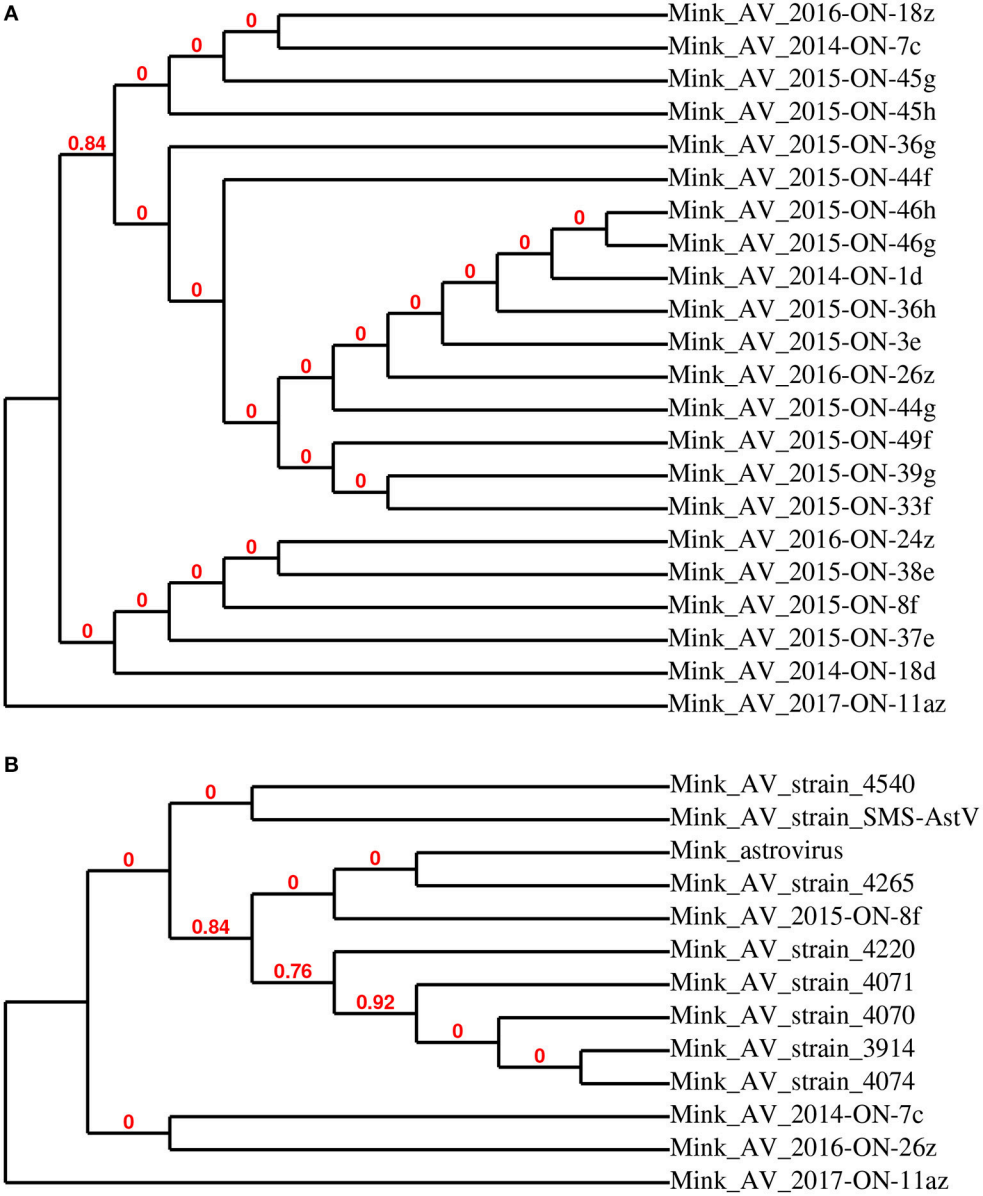
Phylogenetic relationship of astrovirus RdRp partial sequences detected in mink fecal samples **(A)**. Representative strains AV 2014-ON-7c, 2015-ON-8f, 2016-ON-26z, and 2017-ON-11az from this study were used for phylogenetic analysis with closely related astrovirus strains **(B)**.

The cladogram constructed for 23 astrovirus partial RdRp sequences demonstrates clustering into four groups where mink astrovirus (AV) 2017-ON-11az was found to be least related to other detected sequences (Figure 1A). Representative sequences (mink AV 2014-ON-7c, 2016-ON-26z, 2015-ON-8f, and 2017-ON-11az) from each group were compared to mink astrovirus RdRp sequences available in GenBank (Figure 1B). Mink AV_2014-ON-7c, AV_2016-ON-26z, and AV_2015-ON-8f were most closely related to nine previously reported mink astrovirus sequences (>90% identity, NCBI BLAST). Of the representative sequences, mink AV_2017-ON-11az was most distantly related to all sequenced samples and the existing GenBank sequences (<90% identity).

**Figure 2:**
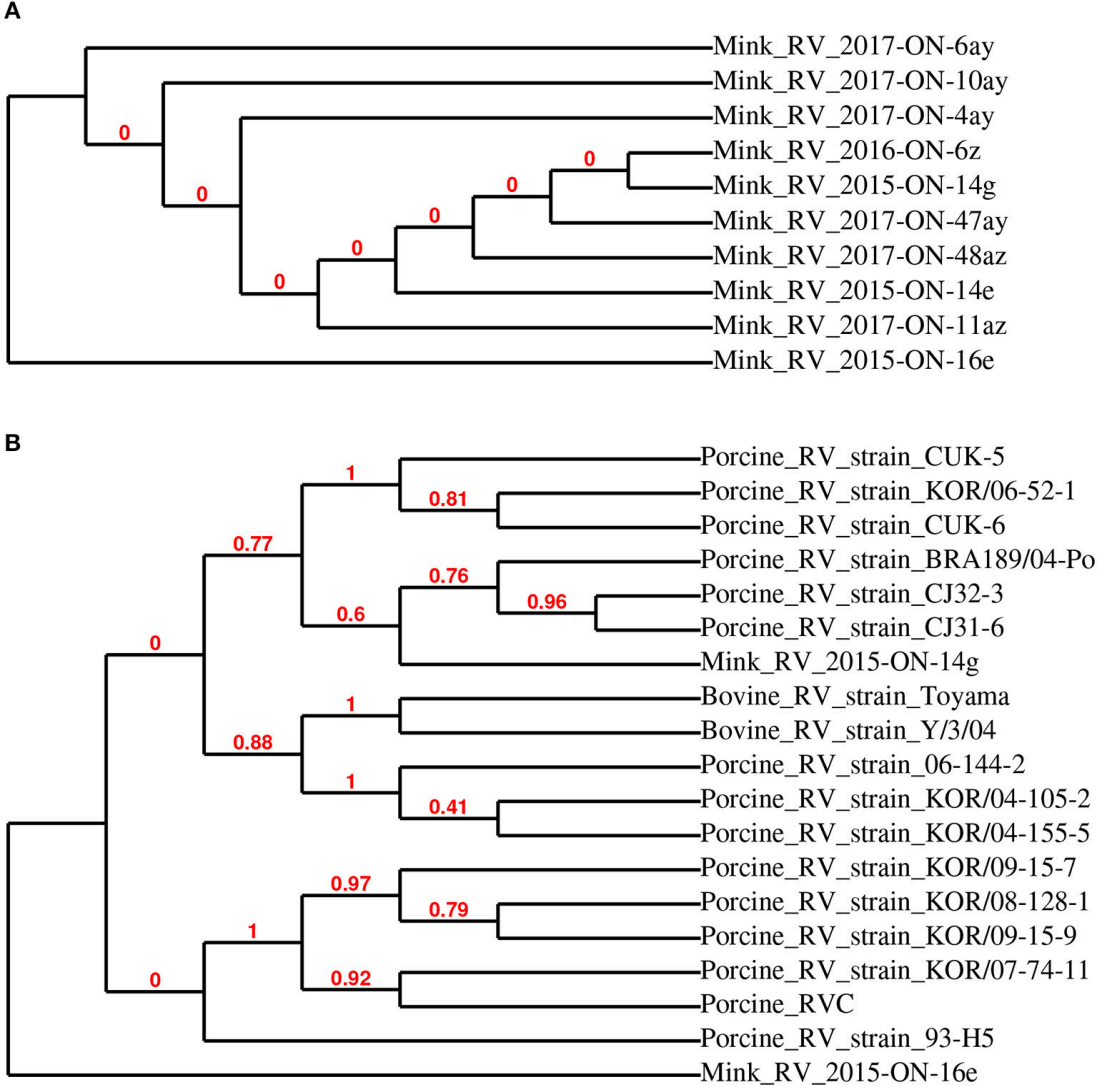
Phylogenetic relationship of detected rotavirus VP6 partial sequences detected in mink fecal samples **(A)**. Representative strains RV 2015-ON-14g and 2015-ON-16e from this study were used for phylogenetic analysis with closely related rotavirus strains **(B)**.

The 10 sequenced RVC samples clustered into two groups (Figure 2A), of which mink RVC 2015-ON-14g and 2015-ON-16e were used as representative sequences for further phylogenetic analysis. When compared to the VP6 gene of 17 other closely related rotavirus C strains, mink RV 2015-ON-14g was most closely related to swine rotavirus C strains BRA189/04-Po (JF810442), CJ32-3 (AB889517), and CJ31-6 (AB889516) (>98% identity). Mink RV_2015-ON-16e was the most distantly related to all analyzed sequences (<95% identity) (Figure 2B).

**Figure 3:**
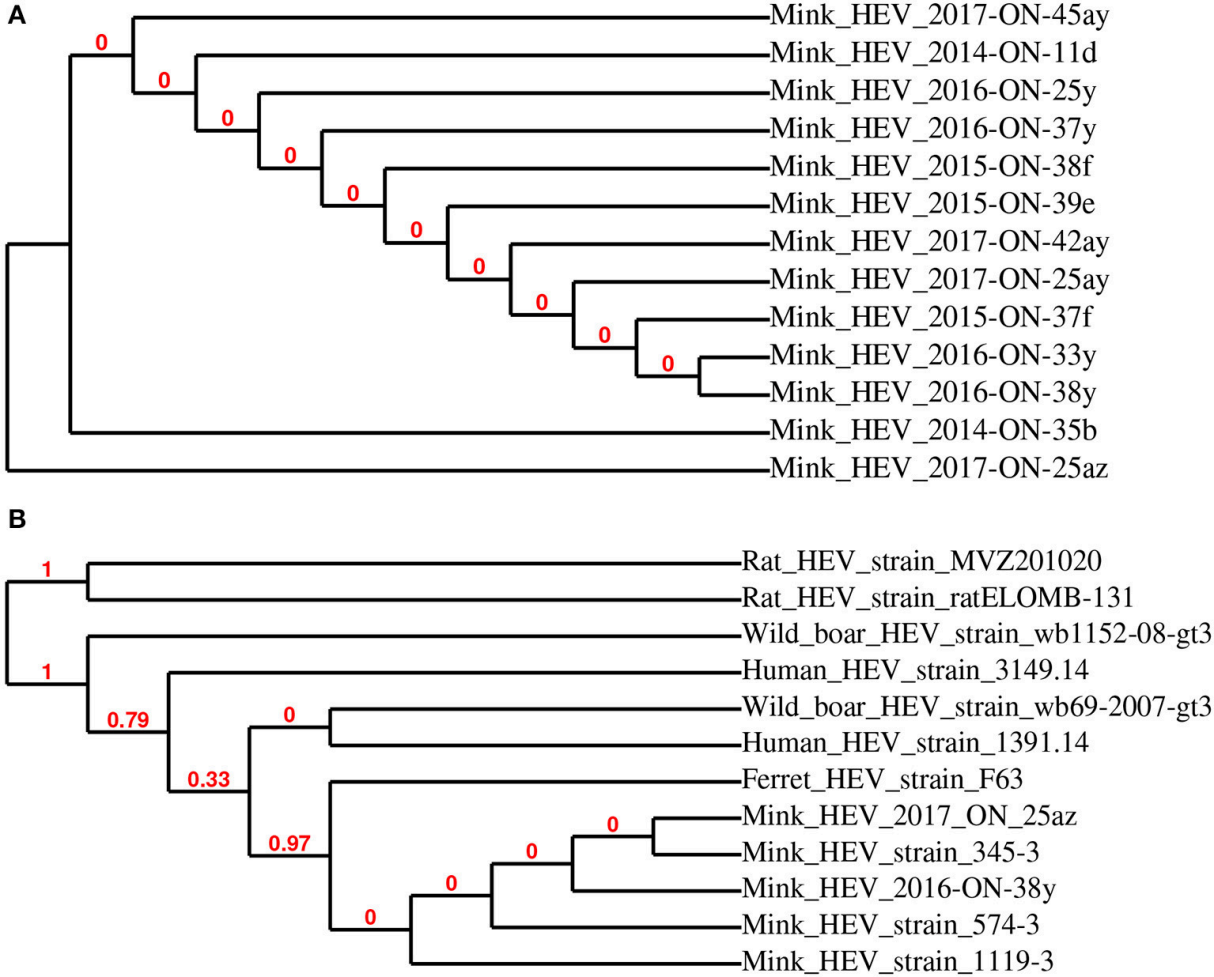
Phylogenetic relationship of mink hepatitis E virus (HEV) RdRp partial sequences detected in mink fecal samples **(A)**. Representative strains HEV 2016-ON-38y and 2017-ON-25az from this study were used for phylogenetic analysis with closely related HEV strains **(B)**.

**Figure 4:**
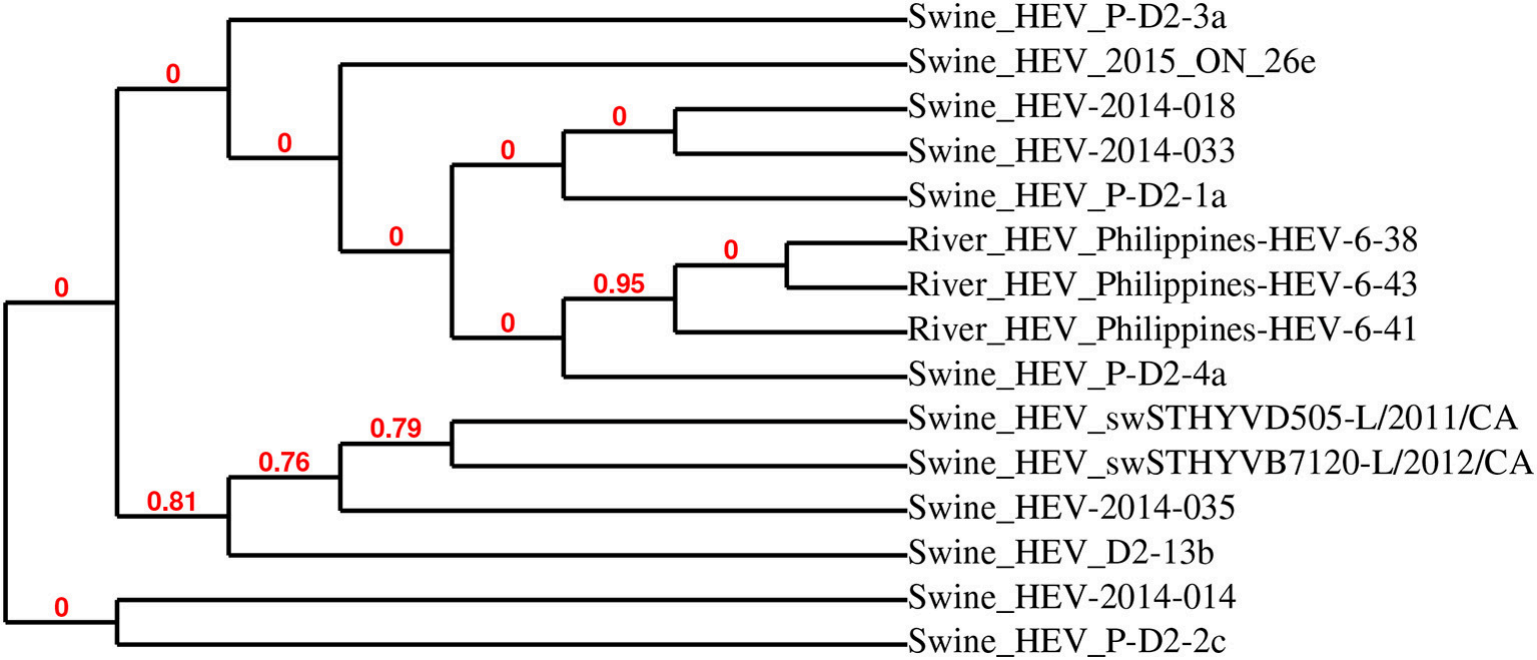
Phylogenetic analysis of detected swine hepatitis E virus (HEV-2015-ON-26e) open reading frame 2 (ORF2) sequence and closely related viruses. HEV-2015-ON-26e was most closely related to swine HEV ORF2 sequences detected in fecal samples collected from Canadian swine herds.

The sequences of the 13 HEV-positive samples were analyzed, and they clustered into two distinct groups (Figure 3A). Further phylogenetic analysis of representative samples mink HEV_2016-ON-38y and HEV_2017-ON-25az with 10 other closely related HEV strains showed that the representative sequences were highly related to mink HEV strains 345-3 (KC802092), 574-3 (KC802093), and 1119-3 (KC802090) (>98% identity), and were also closely related to ferret HEV strain F63 (LC177792) (>86% identity) (Figure 3B). Figure 4 demonstrates the relationship between the sequenced swine HEV positive sample (swine HEV_2015_ON_26e) and closely related swine HEV strains P-D2-3a, 2014-018, 2014-033, P-D2-1a, P-D2-4a (KT778281, KX530975, KX530980, KT778280, respectively) (>93% identity), which were isolated from Canadian swine herds. Swine HEV_2015_ON_26e also clustered closely with HEV strains Philippines-HEV-6-38,−41, and−43 (KF546259, KF546258, KF546257) (93% identity), which were isolated from a river in the Philippines.

## Discussion

This study aimed to assess the prevalence of astrovirus, RVC, and HEV in the feces of Canadian commercial mink. Astrovirus and HEV RNA positive samples were found to be the most prevalent, although positive sample numbers were modest. Overall, higher numbers of pooled fecal samples with detectable viral RNA were identified in the feces of mink kits compared to adult female mink (2014–2015), which is consistent with previous mink astrovirus and HEV research (15, 17). Although viral RNA detected in the fecal samples may not represent active infections, these results indicate that astrovirus and mink HEV RNA was detected in more kits samples compared to adult female samples. In the case of mink astrovirus, these results further demonstrate the need to monitor astrovirus shedding as a potentially important agent in pre-weaning diarrhea (15). Swine HEV was only detected in one sample of the 527 tested, however it must be noted that, due to random sampling of only a small fraction of animals on farm, these results may not be fully representative of true swine HEV prevalence. The detected sequence was also highly related to other strains previously detected in Canadian swine herds, and could be the result of poor biosecurity on farm in feed practices or contamination from commercial animals in proximity to the mink pens (1). According to farm status results, it was common for an astrovirus positive farm to remain positive for two consecutive years, while this event was less common for RVC and HEV positive farms. Only one farm (astrovirus RNA positive) remained positive for three consecutive years. Farms likely did not stay positive for more than two consecutive years due to the high turnover of breeding stock (i.e., most adult females are only used for two breeding cycles in Canada).

A higher number of astrovirus positive samples from the summer cohort is consistent with previous studies showing an increased likelihood of enteric and other infections in the summer (18–20). There was an apparent decrease in the number of astrovirus RNA positive samples (1% of total samples in 2017 compared to 6–25% of total samples in years 2014–2016). For astrovirus, these patterns may not be fully representative of the extent of on-farm infection, since no kit samples were collected in the winters (2016 and 2017) and the majority of astrovirus RNA positive samples from the summer (2014 and 2015) were collected from kits. Sequences of astrovirus-positive fecal samples were highly similar between 2014 through 2016; however, the only astrovirus RdRp sequence detected in the 2017 cohort (mink AV 2017-ON-11az) was phylogenetically distant from sequences obtained previously. The same sequence was also distantly related to nine other enteric mink astrovirus strains detected in this study, with only short segments of the sequenced gene were closely related to previously reported astroviruses.

The increased number of HEV infections in kits may not be directly associated with any specific clinical signs, but could contribute to the severity of disease states caused by co-infection of other bacterial or viral agents (1). Similar to astrovirus, samples from the summer season had slightly higher numbers of HEV RNA positive samples (11% of total samples) compared to samples from the winter (5% of total samples), which may also be attributed to the absence of kit fecal samples in the winter season cohort. The sequenced HEV positive fecal samples in this study were highly related to three other reported mink HEV strains (345-3, 574-3, and 119-3), and also closely related to ferret HEV strain F63. This suggests that the detected HEV strains are primarily mink-specific; the risk of zoonotic transmission of mink and ferret HEV strains are currently unknown (1, 24). The detected swine-like HEV sequence was most closely related to previously identified HEV strains from Canadian swine herds, which may be due to the diet of commercial mink, which is a mixed diet that may consist of pork, poultry and fish products (1, 25). Previous studies have also found a strong correlation between viruses shed in the fecal matter and the diet of animals (1, 25–27).

Although RVC RNA was not highly prevalent in the tested samples, there was an increase in the prevalence of RVC RNA positive samples across the 4 years of the study (8% of total samples in 2017 compared to 0–3% of total samples in years 2014–2016). The detected partial sequences of the RVC VP6 gene were most closely related to swine RVC, which is known to cause diarrhea in swine (8). Little is currently known about mink-specific rotavirus, and it is unclear if swine RVC could cause enteritis in mink. The presence of this swine RVC is also speculated to be related to the mink diet.

Although the results of this study have shown low to moderate levels of astrovirus, RVC, and HEV RNA detected in the feces of clinically healthy Canadian commercial mink, further comparisons between healthy and diseased mink are needed to provide conclusive evidence of the role of these viruses in mink gastrointestinal health. It must be noted that the on-farm prevalence of astrovirus, RVC, and HEV may be higher than those reported in this study, as the pooled fecal samples used for virus RNA detection represent only a small fraction of the animals. Further, it would be interesting to correlate Aleutian disease status on-farm with shedding of these three viruses to determine whether viral co-infections outside of those discussed here would have an impact on mink health. Since these viruses were detected in clinically healthy mink, we speculate that co-infection of these viruses with other viruses, bacteria, and fungi may increase the risk of enteritis. On-farm biosecurity and personal hygiene are poor to low on most Ontario commercial mink farms (13), giving rise to concerns regarding potential cross-species transmission of viruses shed from the feces of apparently healthy mink, especially given recent reports of disease from other species (1, 5, 6, 9, 10). Phylogenetic analyses of sequences showed that although most detected sequences are highly related to previously reported viral genomes, some sequences may be considered divergent and should be further investigated. It must be noted that although the MLE method provided the best estimate of phylogeny, some nodes within the trees are not supported by resampling methods, as indicated by bootstrap values of 0. While this study focused on the detection of astrovirus, RVC, and HEV RNA in fecal samples, a complete virome study would not only allow for further understanding of the viruses shed from healthy commercial mink, but also a better assessment of other viral agents that may be involved in mink health.

## Author contributions

PT, BT, and EN conceived of the work and prepared the grant. X-TX, BT, and RM conducted the work and analyzed the data. X-TX, EN, and PT co-wrote the paper, and all authors contributed to manuscript review.

### Conflict of interest statement

The authors declare that the research was conducted in the absence of any commercial or financial relationships that could be construed as a potential conflict of interest.

## Abbreviations

cDNA: complementary DNA
DEPC: diethyl pyrocarbonate
FDR: false discovery rate
HEV: hepatitis E virus
MiAstV: mink astrovirus
NCBI: National Center for Biotechnology Information
ORF2: open reading frame 2
PCR: polymerase chain reaction
RdRp: RNA-dependent RNA polymerase
RNA: ribonucleic acid.
