# Supplementary material for: Detection of Astrovirus, Rotavirus C, and Hepatitis E Viral RNA in Adult and Juvenile Farmed Mink (Neovison vison)
Source: Front Vet Sci. 2018 Jun 19;5:132. doi: 10.3389/fvets.2018.00132 (PMC6020771; doi:10.3389/fvets.2018.00132)
Supplement: Supplementary file 1 [file Table_1.docx]

**Supplementary Table 1.** Accession numbers of representative astrovirus (AV), rotavirus (RV), and hepatitis E virus (HEV) sequences detected in fecal samples collected from commercial mink in Ontario.

| Representative sequence | Accession number |
| --- | --- |
| AV-2014-ON-7c | MH282878 |
| AV-2015-ON-8f | MH282879 |
| AV-2016-ON-26z | MH282880 |
| AV-2017-ON-11az | MH282881 |
| RV-2015-ON-14g | MH282882 |
| RV-2015-ON-16e | MH282883 |
| HEV-2015-ON-26e | MH282884 |
| HEV-2016-ON-38y | MH282876 |
| HEV-2017-ON-25az | MH282877 |
